# Predictors of Caregivers’ Satisfaction with the Management of Children with Autism Spectrum Disorder: A Study at Multiple Levels of Health Care

**DOI:** 10.3390/ijerph16101684

**Published:** 2019-05-14

**Authors:** Nik Aida Nik Adib, Mohd Ismail Ibrahim, Azriani Ab Rahman, Raishan Shafini Bakar, Nor Azni Yahaya, Suria Hussin, Wan Nor Arifin Wan Mansor

**Affiliations:** 1 Department of Community Medicine, School of Medical Sciences, Universiti Sains Malaysia, 16150 Kubang Kerian, Kota Bharu, Kelantan, Malaysia; eyedasyukran@gmail.com (N.A.N.A.); azriani@usm.my (A.A.R.); 2 Department of Psychiatric, School of Medical Sciences, Hospital Universiti Sains Malaysia, 16150 Kubang Kerian, Kota Bharu, Kelantan, Malaysia; raishanshafini@usm.my; 3 Department of Pediatric, Hospital Raja Perempuan Zainab II, 15200 Kota Bharu, Kelantan, Malaysia; drnorazni@moh.gov.my; 4 Department of Psychiatric, Hospital Raja Perempuan Zainab II, 15200 Kota Bharu, Kelantan, Malaysia; drsuria@moh.gov.my; 5 Unit of Biostatistics and Research Methodology, School of Medical Sciences, Universiti Sains Malaysia, 16150 Kubang Kerian, Kota Bharu, Kelantan, Malaysia; wnarifin@usm.my

**Keywords:** ASD, caregiver, satisfaction, multiple levels of health care

## Abstract

Background: Caregivers are the initial gatekeepers in the health care management of children with autism spectrum disorder (ASD). Methods: This cross-sectional study aimed to determine the factors associated with caregivers’ satisfaction with different levels of health care services in managing children with ASD in Kelantan. The satisfaction scores of 227 main caregivers of confirmed ASD children were assessed with a modified Parent Satisfaction Scale (PSS) questionnaire. Results: The analysis showed that caregivers who waited longer for a doctor’s consultation in primary care had a reduced PSS score, whereas caregivers who were satisfied with the waiting time in primary care had higher PSS scores. At the secondary care level, caregivers who possessed at least a diploma had reduced PSS scores, whereas caregivers who were satisfied with both doctors’ consultation times and occupational therapy appointments had higher PSS scores. At the tertiary care level, caregivers with an underlying medical problem and who had children undergoing occupational therapy for two months or more had reduced PSS scores. Nevertheless, the analysis showed that caregivers who were concerned with their children’s sleeping problems, who had been informed about parental support, who were satisfied with speech and occupational therapy appointments, who were satisfied with waiting times at tertiary care clinics, and who were satisfied with their doctor’s knowledge and experience had higher PSS scores. Conclusions: This study elucidated the importance of understanding caregivers’ satisfaction in attaining care for their ASD children and highlighted the need to promote factors that would increase caregivers’ satisfaction with current ASD services.

## 1. Introduction

Caregivers of children with autism spectrum disorder (ASD) serve as gatekeepers for accessing available health care services. However, they face a complex health care management system when seeking the diagnosis and treatment of and therapies for their children with autism. The satisfaction level of caregivers of children with ASD regarding health care services is important to determine, as doing so would provide evidence of whether health care providers should meet caregivers’ expectations to ensure the continuation of desirable care [1]. It is also essential in determining the accessibility and equality of health care delivery that is widely accepted and sustained in various healthcare settings [2].

Globally, access to health care services and support for children with ASD are still insufficient [3]. The nature of ASD demands a wide range of health care services comprising both medical and rehabilitative care. The health care management of children with ASD has often been described as unsatisfactory by their caregivers [4]. Delays in obtaining diagnosis [5,6,7], the number of professionals involved [8,9], waiting times for doctor consultations [10,11], consultation times with health care providers [9,10], knowledge and experience on the part of health care providers [12], the frequency of therapy appointments [10,13], and the amount of support and explanation provided during and immediately after diagnosis [5,13,14,15] have all been studied in relation to understanding caregivers’ experiences of the process of health care management of children with ASD.

Although a local study conducted among children aged 18 to 26 months revealed that one in every 625 Malaysian children has ASD [16], many ASD cases remain unrecognized. Medical and educational practitioners have been witnessing a rising number of children with speech delays and communication difficulties, a trend which requires further assessment by trained clinicians [17] and which supports the notion that the true prevalence rate is probably much higher.

Malaysia generally has an efficient and widespread system of health care, operating a dual-tiered system of health care services: a government-led and funded public sector, and a thriving private sector creating a dichotomous yet synergistic public-private model [18]. The medical care services provided by the public health care facilities comprise of three levels: primary, secondary, and tertiary care through a wide network of health clinics and hospitals. These include outpatient and inpatient care services ranging from primary care (health clinics) to the advanced medical care at tertiary care options (specialist hospitals) [19]. Primary care is the thrust of Malaysian health care system and acts as a national referral system. The services provide greater equity, accessibility and better utilization of the resources. Primary care service was supported by secondary care services, which are devolved and regionalized tertiary care services [20]. The primary care services comprise of outpatient department as the first point of contact. The services run by a medical officer and visiting or and Family Medicine Specialist and or Occupational Therapist (OT) and or social welfare depending on the type of primary care [19]. Secondary care is where most people end up when they have a medical condition to deal with that can’t be handled at the primary care level. Secondary care services comprise of general medicine, pediatrics, obstetrics and gynecology and rehabilitation therapy. The services were run by medical officers, occupational therapists and visiting specialists from different specialties. Once a patient is hospitalized and needs a higher level of specialty care within the hospital, he or she needs to be referred to tertiary care [19].

The management of children with ASD varies according to type of health care services. The clinical management of children with ASD consists of routine developmental assessment and screening, followed by diagnosis confirmation by specialist and multidisciplinary assessment. The routine developmental assessment and screening was conducted at primary and secondary care. However, due to placement of the therapist, the intervention for ASD children was conducted at secondary care and tertiary care. With regard to the suggestion to combine of both primary and secondary care, we think separation is better as each level of care provides different management for children with ASD not only in terms of assessment and referrals. With separate analysis, it can also provide an overview of ASD management at different levels of the health care system in Malaysia and indicate where is the gap for improvement [19].

A thorough clinical assessment of children with ASD consists of an integrated, detailed developmental assessment with caregiver interviews and screenings followed by diagnostic confirmation by a specialist and a multidisciplinary assessment. Routine developmental assessments and screenings are conducted at primary care (health clinics) and secondary care centers (district hospitals), while diagnostic confirmation is conducted at tertiary care centers. All children in Malaysia must undergo a screening process using a specifically tailored checklist called the Modified Checklist of Autism in Toddlers (MCHAT) between the age of 18 months and 36 months. Children with low MCHAT scores are referred for further evaluation and verification of ASD in tertiary care [21].

Data from Asian countries concerning caregivers’ perceptions of and satisfaction with the management of ASD in the health care setting are extremely limited. Yet caregivers’ perceptions and satisfaction are crucial to understand, as they may help to improve health care services, owing to their substantial role in the children’s overall development.

Caregivers of children with ASD are reported to have variable experiences regarding the impact of health care procedures and management of their children. The majority of caregivers have noted that service provisions do not meet their expectations and have claimed that the management provided by the health care system is a significant source of stress [21,22,23]. As of now, research on caregivers’ stress and satisfaction with the health care management of children with ASD has mostly been confined to developed countries and regions, such as Canada, the US, and Europe [21,24,25].

To the best of the researcher’s knowledge, no study has been performed to assess caregivers’ satisfaction with the health care management of ASD. Out of 79 publications related to ASD in Malaysia, none have discussed this issue. Rather, most of these studies evaluated the child’s outcome while ignoring the caregivers’ perceptions, particularly their perceptions of health care services like accessibility, difficulties dealing with the health care system, level of satisfaction with the services provided, and need for post-diagnosis support [17].

In response, this study addressed caregivers’ perceptions of health care services received throughout the process of obtaining a diagnosis of ASD in their children. The findings of this study delineate some important aspects of the evaluation of health care management of ASD by taking into consideration caregivers’ opinions regarding the services provided.

According to the latest National Health System Review (NHSR), a study on caregivers’ satisfaction is highly recommended and essential for improving the efficiency of the current services [26]. Therefore, input from caregivers about health care management is crucial for evaluating the current ASD services.

## 2. Materials and Methods

### 2.1. Study Setting and Participants

A cross-sectional study, starting in February 2018, was conducted for two months among caregivers of ASD children who received treatment from four tertiary care clinics in Kelantan: The Hospital Raja Perempuan Zainab II, Hospital Universiti Sains Malaysia, Hospital Kuala Krai, and Hospital Tanah Merah. 

This study (what was practiced in Malaysia) involved three levels of care whereby those who were managed at primary and secondary care need to be referred to tertiary care for confirmation of diagnosis. While we did assess their satisfaction level starting from primary care not all of them proceeded through all three levels of care. Some of them for example started in primary care and were later referred straight to tertiary care. Some were referred to secondary care before proceeding to tertiary care, or initially went to secondary care and were later referred to tertiary care. Another option was that they went straight to tertiary care. Thus, we tried our best to include all caregivers who were eligible for our study. 

In our study only trained doctors, specifically pediatricians and psychiatrists made diagnoses at a tertiary level, while assessments were done by the multidisciplinary team (speech, occupational therapist and child psychologist) rather than only by doctors. Intervention was carried out by speech and occupational therapists.

The sample size requirement in this study was calculated using G*Power software version 3.1.9.2 (Department of General Psychology, Düsseldorf University, Germany, 2009) for linear regression [27]. For α = 0.05, power = 0.8, effect size = 0.15 (medium) and 24 predictors and total number of tested predictors = 12, the minimum sample size required was n = 128. This was inflated to n = 154 after taking into account a 20% dropout rate. In this study, we have a total of 412 caregivers who currently being followed up at tertiary care. We randomly selected 227 caregivers out of whom 126 had been seen at primary care, 46 had a history of being reviewed at secondary care and 227 were seen at tertiary care. Thus, there was no issue of caregivers who used primary and secondary services without their views were not sought. With regard to the fees payable by caregivers, they only need to pay RM1 (USD 0.24) to get all the services required for their child.

A stratified random sampling method was employed to select respondents from the identified list of children with ASD who attended follow-ups. We utilized a stratified sampling method in which the stratification variable was the health care setting. The sample size per strata was proportionate to the list of existing ASD patients treated at each setting. 

Main caregivers who were illiterate and/or known to have an underlying psychiatric problem were excluded from the study. In addition, children for whom the diagnosis was not yet confirmed during the study period were also excluded. We did not include caregivers who did not follow up at tertiary care in this study. The adult literacy rate of Malaysia has increased from 69.5% in 1980 to 94.6% in 2018 [28]. Unfortunately, we do not have data on the same matter in the state of Kelantan as well as at the district level. However, most of the caregivers claim to have good literacy. Only less than 1% was found to have poor literacy due to a poor academic background. Those caregivers who fulfilled the study criteria were included in our study criteria.

The consenting caregivers were given a Malay version of the Parent Satisfaction Scale (PSS-M) questionnaire, including a pro forma. In total, there were 74 questions that needed to be answered by the caregivers. They were given about five minutes to read the consent form before they gave their consent. Then, the participants were given another 25 min to complete the set of questionnaires.

A main caregiver was defined as a person who is primarily responsible for the development of a child and who is most involved in their health management, including hospital visits, consultations with specialists, and follow-ups for all kinds of clinical interventions. Therefore, main caregivers may be biological parents or individuals charged with caring for a child since his or her birth. As for the current study, comorbidity is defined as those who have or not have any medical problem or other disorders apart from ASD. The waiting time is referred to as the point of registration until they were called for a meeting with the treating doctor. In current practice, a patient only has an appointment with only one treating doctor per visit. Waiting time at each clinic for different levels of health care is not the same. Regardless of the level of health care, the measurement waiting time to be seen by the treating doctor is still the same. Thus, the definition for our waiting time as such compared to few other studies which have a different meaning, for instance, waiting time to receive the confirmation of ASD starting from meeting with their professional.

### 2.2. Parent Satisfaction Scale, Malay Version (PSS-M)

The questionnaire used in this study was developed by Gerkensmeyer and Austin [29]. It was then translated into Malay and validated by Nik Adib, Ibrahim [30] among 110 caregivers of children with ASD in Kelantan. The one-dimensional PSS-M is a self-administered questionnaire consisting of 11 statements or items. Each statement has five Likert-scale responses, ranging from one to four with score 0 representing strongly disagree and score 4 representing strongly agree. The total 11-items of the PSS-M score ranged between 0 to 44. Higher scores indicate a higher level of satisfaction with the interpersonal relationship between a caregiver and a professional. In this study, respondents were asked to choose one best response for each statement. The PSS-M showed good reliability, and its internal structure fit the model well (Standardized Root Mean Square (SRMR) = 0.056, Root Mean Square Error of Approximation (RMSEA) = 0.046, Comparative Fit Index (CFI) = 0.97, Tucker Lewis fit Index (TLI) = 0.96), with good construct validity and excellent internal consistency reliability: rho 0.851 and a 95% Confident Interval (CI) (0.81, 0.89).

### 2.3. Statistical Analysis

In the current study, all data were entered using SPSS software version 24. Data were checked and cleaned. Preliminary data screening was done for missing values or possibly incorrect data entry. Corrections or completions for missing values were carried out by reviewing the information recorded in the pro forma. Missing data for specific, individual items in the study were excluded from analysis. Generalized Linear Model (GLM) was applied in this study using identity link function for linear-response data. Dummy variables were generated for categorical variables within GLM framework. The regression analysis was run using R version 3.3. (R Foundation for Statistical Computing, Vienna, Austria, 2013) “using *GLM* function” to identify the predictors of caregivers’ satisfaction with the health care management of children with ASD. The analysis was done involving the four tertiary cares. Unfortunately, we do not include those variables that not significant in the linear table as the type of tertiary care has no effect on the model. 

### 2.4. Ethical Consideration

The approval for this research was obtained from the Human Research Ethics Committee of University Sains Malaysia (JEPeM Code: USM/JEPeM/17110600) and National Medical Research Register (NMMR) Malaysia (NMMR-17-2732-38655). The confidentiality of the data had been strictly maintained. Only the author and supervisors had access to the data. Later, the reporting and publications were carried out with no respondents’ names mentioned.

## 3. Results

### 3.1. Socio-Demographic Characteristics of the Caregivers

The mean (SD) age of caregivers was 38.91 (8.26) years. Majority of the caregivers were female (74.9%), and the majority had education levels at the Diploma holder level and above (63.9%). Table 1 shows the socio-demographic characteristics of the caregivers.

### 3.2. Socio-Demographic Characteristics of the ASD Children 

The mean (SD) age of ASD children was 7.45 (3.54) years. The majority of them were boys (82.8%), with co-morbidity (50.2%) and have registered with Social Welfare Department (58.6%). The summary of the findings is presented in Table 2.

### 3.3. Management of Children with ASD and Level of Caregivers’ Satisfaction (n = 227)

The mean (SD) of the time taken for diagnosis of ASD was 1.24 (0.95) years. The mean (SD) number of doctors consulted before diagnosis was confirmed was 3.45 (1.65), the majority of whom were psychiatrists (56.4%). Among 227 respondents, 126 had sought treatment at the primary care level before immediately being referred to tertiary care, while only 46 had sought treatment at the secondary care level before being referred to tertiary care for diagnostic confirmation and further management. Thus, the results show respondents’ satisfaction levels at different levels of care. 

### 3.4. Level of Satisfaction with the Relationship with Professionals at Different Health Care Facilities

The mean (SD) of the PSS-M total score at primary care was 26.55 (7.16), whereas the mean (SD) of the PSS-M total score at secondary care was 30.06 (6.47). The mean (SD) of the PSS-M total score at tertiary care was 31.79 (6.25). A summary of the mean scores of satisfaction among caregivers of children with ASD at different health care facilities is illustrated in Table 3.

### 3.5. The Predictors of Caregivers’ Satisfaction

In attempting to understand the factors that could predict caregivers’ satisfaction with the health care management of children with ASD at different levels of the health care setting, all the variables in the study and the PSS scores were entered into a regression analysis, as these factors were significantly associated with caregivers’ satisfaction. There were six significant predictors of caregivers’ satisfaction at primary care, three at secondary care, and eight at tertiary care. A summary of all the predictors of caregivers’ satisfaction at each type of health care facility is displayed in Table 4, Table 5 and Table 6.

Final model equation of caregiver satisfaction with health care management of ASD at primary care: 22.17 + (3.34 * caregiver with household income between RM 5001–RM 8000) + (−2.33 * presence of hypersensitive child) + (−6.78 * waiting time for doctors’ consultation more than 2 h) + (−2.25 * consulted doctors more than 3 times) + (3.88 * satisfied with waiting time) + (2.71 * satisfied with consultation time with doctors).

Final model equation of caregiver satisfaction with health care management of children with ASD at secondary care: 26.097 + (−5.99 * caregiver education) + (4.05 * satisfied with occupational therapy appointment) + (5.93 * satisfied with consultation time with doctors). 

Final model equation of caregiver satisfaction with health care management of children with ASD at tertiary care: 20.83 + (−6.09 * caregiver with medical problem) + (1.66 * child with sleeping problem) + (3.11 * caregiver offered parent support group) + (−5.23 * frequency occupational therapy twice monthly or more) + (1.66 * satisfied with frequency of speech appointments) + (3.82 * satisfied with frequency of occupational therapy appointments) + (2.55 * satisfied with waiting time) + (4.39 * satisfied with doctor’s knowledge and experience).

The variance accounted by the regression (R^2^) ranged from 32.92% for tertiary care to 50.65% for secondary care. This indicates that there are other factors that influence the satisfaction score but were not investigated in this study. It is recommended to do further study to investigate the remaining factors that might influence the satisfaction score.

## 4. Discussion

This study aimed to determine the predictors of caregivers’ satisfaction with the health care management of ASD at all health care settings in Kelantan. The health care management of ASD includes caregivers’ experiences prior to diagnosis, the process of diagnosis, and the post-diagnosis support caregivers of children with ASD received. A total of 227 caregivers completed the questionnaire. The current findings represent the first report on the satisfaction of caregivers of ASD children with health care management in different health care settings in Malaysia. Thus, these findings have the potential to inform the manner in which the elements we highlighted must be improved by health care providers in managing children with ASD at all levels of health care based on caregivers’ perspectives. 

In this study, the majority (64.3%) of caregivers chose to visit primary care to seek opinions about their child’s problem. They were then referred to tertiary care for suspected ASD diagnosis and further management of their child.

### 4.1. Management of Children with ASD and Caregivers’ Satisfaction at Different Levels of Health Care 

#### 4.1.1. Prior to Diagnosis

Caregivers were well aware of problems related to their child’s development at an average age of 2.66 years. This finding is similar to those reported by Moh and Magiati [9] in Singapore. By comparison, in the UK, southeastern Europe and Saudi Arabia, caregivers first reported noticing developmental delays earlier, around their child’s second birthday [12,14,17]. The possible reason why Malaysian caregivers might recognize development delays later is that, in such collectivistic cultures, encroaching on other people’s business is discouraged. Therefore, caregivers may be less likely to identify problematic behaviors as a cause for concern [9]. Further, families with ASD children are also exposed to additional financial burdens, including significant extra health care management costs as well as non-medical expenses, such as school services and therapies [23,31,32,33]. Moreover, the increased number of families where both caregivers are working may prevent the vigilant monitoring of early warning signs, as their children are often sent to nurseries. 

Caregivers in our study first noticed delays in speech and social development, followed by ritualistic behaviors and the failure to develop age-appropriate learning abilities. Caregivers who note social development concerns earlier are also more likely to receive an earlier diagnosis for their children [33]. Given that Malaysian caregivers noticed difficulties in their children somewhat later, efforts to increase caregivers’ knowledge about child development could potentially help them understand what to expect and how to provide for their children’s needs in early childhood. As such, caregivers would likely seek screening services if they noticed any atypical developments in their children, paving the way for earlier diagnosis.

The majority of caregivers in this study had a moderate level of household income, a finding consistent with other studies [9,34,35]. It seems that caregivers with more economic resources and higher educational qualifications may be more vigilant in noticing their children’s developmental difficulties earlier, regardless of the severity of these difficulties. Thus, parents from lower socio-educational backgrounds may be an important group to target in terms of education and awareness of child development and early warning signs of ASD.

#### 4.1.2. Process of Diagnosis

In the current study, caregivers were found to have waited on average more than one year to start contacting a health care professional and on average nearly 1.3 years to receive a formal ASD diagnosis, suggesting a delay of more than two years. This delay is similar to that experienced by caregivers in the UK [12] and more than one-half of that experienced by caregivers in Saudi Arabia [14]. The delay in seeking help reflects a lack of knowledge among certain caregivers about the importance of early diagnosis and intervention. The average age of children’s diagnosis is 5 years old, which is similar to that in the UK and Canada [8,12] but longer than that in Singapore, where children have been reported to be diagnosed at a mean age of 3.4 years [9]. Similarly, in France, diagnosis typically occurs prior to the child’s fourth birthday [36]. The actions of parents associations have caused a growing awareness of the efforts that must be made in the field of autism in France and Singapore. As for the current study, one-half of the children were diagnosed immediately upon their first visit, which reflects the implementation of good clinical practices and an efficient system; nevertheless, earlier and timelier diagnosis does not result. This is because each child is unique, and some children present with mild ASD symptoms. Therefore, multi-disciplinary team observations and input are required before any confirmation of ASD diagnosis.

Caregivers mainly consulted on average three to four professionals of various fields before obtaining a formal ASD diagnosis. This number is slighter smaller than the four to five professionals consulted in Canada [8] and the US [11]. The number of doctors consulted varies according to the child’s spectrum and the health care management of ASD at each health facility. One-half of the children with ASD in the current study were associated with comorbid symptoms; therefore, the number of professionals consulted is actually fewer when compared to Singapore [9].

#### 4.1.3. Support Post-diagnosis

The findings from the current study point to the need to improve the health care management of ASD, specifically post-diagnosis support. That is, almost all caregivers received no information about or referral to parent support groups. More than one-half claimed to receive no help in terms of information concerning their child’s overall condition or suggestions for appropriate schools and education, similar to UK caregivers interviewed by Osborne and Reed [6] and US caregivers [34]. Caregivers in both these and the current study clearly prioritize and value being given information that would help them make sense of their child’s core presenting difficulties, information about treatment options, and information about the best interventions for their child.

### 4.2. Level of Satisfaction with the Relationship with Professionals at Different Health Care Facilities

The total score level of caregivers’ satisfaction with their relationship with professionals rated lowest at primary care and highest at tertiary care. The caregivers rated item 8 at the lowest level, which concerned caregivers’ satisfaction regarding staff informing them about their child’s condition, followed by item 7, which concern the attitude of the professional toward caregivers. This finding is in line with a study conducted by reference [14], where caregivers perceived lower satisfaction with these items. A major step toward increasing caregiver satisfaction with ASD management would be to ensure that diagnosticians and supporting staff have the necessary skills to provide caregivers with sufficient information and know how to be good listeners to enhance the accuracy of the services provided.

### 4.3. Predictors of Caregivers’ Satisfaction with the Health Care Management of Children with ASD

In the current study, caregivers reported dissimilar levels of satisfaction at different types of health care settings. It was found that caregivers with a household income of RM 5001 to RM 8000 had a PSS score of 3.34 at primary care. This finding from Benzoni [33] is in line with Moh and Magiati [14], who found that caregivers with more than a moderate level of household income were associated with earlier recognition of ASD problems and sought treatment earlier; thus, they also reported higher satisfaction with health care services. The current findings, however, contradict those of Liptak, who reported that those families with a household income below the poverty line had less access to services, thus leading to a decrease in caregiver satisfaction levels with the management of children with ASD. The interviewed telephone interviews were conducted among 495 caregivers of children with ASD where one- fifth of them were from poor Latino or Black families. Generally, these ethnicities were found to have worse access to all the observed care parameters. It was revealed many such families are less likely to use doctors’ services, less likely to seek timely medical care, lack of regular care resources, more difficult to get referrals to experts and more likely to have worse health status than children from middle-aged families or high income [34].

Psychiatric comorbidities are common among children with ASD. Such children may experience a broad spectrum of difficulties with attention, behavior, emotion, thought, and health. Having ASD with comorbidities may lead to significant clinical impairment in most children. Common comorbidities among children with ASD include intellectual disability, attention deficit hyperactivity disorder (ADHD), sleep problems, epilepsy, gastrointestinal problems, and problems with motor coordination [17]. The current study found that caregivers who had a hypersensitive child had a reduced PSS score regarding primary care, by 2.33. This result is congruent with research by Perreault, Rousseau [13] in Canada, which showed that caregivers of ASD children with comorbidities, e.g., hypersensitivity and behavioral problems, had lower satisfaction levels toward health care services. On the other hand, caregivers of children with sleeping problems had increased PSS scores, by 1.65, toward specialist centers. This positive association is attributable to health care professionals properly tackling the caregivers’ concerns by prescribing medications, cognitive behavior therapy and sleep training to mitigate their child’s sleeping problems [37]. Consequently, their children became more focused, less irritable, and had fewer tantrums, which in turn improved the overall quality of life for the caregivers’ families and directly influenced caregiver satisfaction with the care received.

The caregivers often consulted a number of professionals before diagnosis of ASD was finally confirmed. The number of doctors consulted varied according to the child’s condition on the ASD spectrum and the health care management received at each health facility. The waiting times between requesting an appointment and being seen at the clinic for doctors’ consultation are within 6 weeks for both pediatrics and psychiatric departments. The current study demonstrated that caregivers who consulted doctors more than three times for diagnosis confirmation had a lower PSS score (2.25) regarding primary care. This finding is supported by Goin-Kochel, Mackintosh [11], who found that caregivers were more satisfied with the diagnostic process when they saw fewer professionals. It seems that the more doctors are visited, the more hassles are faced by caregivers in terms of direct and indirect costs (e.g., waiting times for consultations and rescheduling working hours). With regard to early identification, published clinical practice guidelines recommend that all professionals in health care and pre-schools as well as caregivers be familiar with typical child development and “red flags” relating to how ASD may present in children [38].

Caregivers rely on health care professionals, especially doctors, to provide information and to discuss problems concerning their ASD child. It was found that caregivers who were satisfied with doctors’ consultation times gave higher PSS scores, by 2.71, to primary care and secondary care, by 5.93. This finding is supported by previous studies in which caregivers claimed to be less satisfied with overall services when the health care professional did not take into account their early concerns in a supportive and empathic manner or when shorter durations of consultations with doctors did not allow enough time to discuss issues concerning caregivers’ children [6,9]. Previous studies also revealed that caregivers were concerned with time constraints when meeting with health care professionals [6,15]. It is important that health care professionals review patient records and are knowledgeable concerning ASD before conducting consultations, doing so would allow detailed interviewing regarding current complaints and caregiver concerns, which would in turn be addressed by the doctor, who could provide solutions that best meet the needs of the children.

In the current study, caregivers who waited more than two hours to meet with doctors at primary care had a PSS score that was reduced by 6.78. Caregivers who were satisfied with the waiting time for a doctor consultation showed an incremental PSS score increase of 3.88 at primary care and 2.47 at tertiary care. This finding is in line with study conducted by Moh and Magiati [9]. The authors stated that long waiting times were the most stressful experience for caregivers and the least satisfying aspect of health care services; in addition, caregivers did not feel they had enough time to consult with their doctor and share their worries, thus leaving them frustrated. The possible reason for this situation was a lack of manpower and skillful and trained specialists in the field of ASD. When patients present with typical signs of ASD, it should be easier to confirm that these patients have ASD; however, if the signs are atypical with comorbidities and a mild spectrum, then consultation with an expert is needed and requires more time. This would in turn slow patient turnover and increase waiting times at clinics. In tackling these issues, doctors should be trained to familiarize themselves with the care and proper management of ASD at health care facilities so that the best advice and proper channel of care can be directed to caregivers. The empowerment of caregivers and continuous medical education may be achieved through seminars, workshops, and social media.

The caregivers’ education level was found to be associated with earlier recognition of problems and earlier visits to health care facilities for further investigation [39]. The education level of caregivers has also been shown to be positively associated with satisfaction with the management of children with ASD [14]. This is because more than half of the respondents in reference [14] had degrees and live in major cities. Due to these factors they can get early professional help and receive diagnosis confirmation before the children are 2 years old. Specific ASD intervention was also done earlier. Finally, caregivers were happy to see their children’s social skills and respond better in society in a relatively short period of time [14]. However, in the current study, it was found that the PSS score was reduced by 6 when caregivers held at least a diploma. This finding is congruent with a study by Potter [40]. The majority of caregivers claimed that the information given was inadequate, not tailored, and non-specific to each family who was struggling with a newly received diagnosis. It would seem that the majority of caregivers with a higher education level should have prepared themselves with relevant information regarding ASD. However, if their concerns and information were not delivered to the best according to the needs of caregivers, it might influence their satisfaction toward services.

The frequency of therapies is one of the predictors of ASD prognosis. Intensive behavioral intervention, e.g., speech and occupational therapy, promote better communication skills, improve daily social skills, and result in positive social behaviors [40]. The current study revealed that those caregivers who were satisfied with speech and occupational therapy at secondary and tertiary care had increased PSS scores: 5.93 for occupational therapy at secondary care, 3.94 for occupational therapy at tertiary care, and 1.92 for speech therapy at tertiary care. However, it was found that the the PSS score was reduced by 5.23 when caregivers’ children attended occupational therapy once in two months or more often. This finding is also supported by a study in Canada in which caregiver satisfaction with services increased if they were given better access to rehabilitation [13]. The majority of children with ASD in the current study had speech and occupational therapy appointments monthly or twice monthly depending on their spectrum. With this frequency, caregivers should see immediate benefits of these therapies in their children. This includes an increased ability to integrate in the social environment and a more nurturing and supportive environment for the entire family, thus influencing their satisfaction level toward such services. In addition, a few caregivers also suggested that it would be better if backup therapies were available in the event a therapist was on leave. This is because some caregivers claimed that their child was not undergoing rehabilitation when their therapist went on postnatal leave. Thus, this issue should be taken into consideration by the rehabilitation management team in order to avoid non-compliance and regression in child behavior issues in the future.

Underlying medical problems and, at the same time, being a caregiver for children with ASD are big issues for caregivers, who must provide dynamic caregiving over time and bring their children in for follow-ups at health care facilities. The current study found that caregivers with underlying medical problems showed a reduction in PSS scores by 5.98 regarding specialist centers. This finding is supported by Saloojee, Phohole [41], who highlighted the difficulties experienced by caregivers in complying with their child’s rehabilitation therapy due to their own illness. In this case, it would be better to provide faster services, e.g., to elderly caregivers and caregivers with physical limitations, to reduce their waiting times during busy days in specialist clinics.

Parent support groups could create a meaningful context of exchange to help caregivers reduce social isolation and allow them to access information about children with ASD, as well as to learn advocacy skills and gain confidence in their caregiving role [13,15]. The current study discovered that those caregivers who were offered and informed about parent support groups showed an incremental PSS score increase of 2.66 at tertiary care. Even though most caregivers value support groups, studies conducted by Osborne and Reed [6] in the UK revealed that nearly 40% of caregivers claimed they were not given any information that would improve their understanding of ASD. Another group of caregivers in the UK claimed that even though they did receive support, none of it was organized for the caregivers’ convenience, nor was it appropriate or geared to their needs [12]. The availability of parent support groups is crucial, as such groups create awareness and early detection among caregivers and providers as well as the necessary efforts that must be made in the field of ASD, as demonstrated by the availability of the Autism Parent Association in France [36].

The caregivers relied on their health care professionals to get the best treatment for their children with ASD. Therefore, health care professionals should be well equipped with knowledge and skills related to ASD, since diagnosis cannot be confirmed by an untrained and unspecialized doctor. It was shown that those caregivers who were satisfied with their doctor’s knowledge and experience showed an incremental increase of PSS scores by 4.14 at tertiary care. This finding is consistent with those from previous research on the ability of doctors to provide adequate information and intervention for ASD according to individual needs [42].

Caregiving tasks are a potentially underestimated predictor of satisfaction. Given that fewer caregiving tasks predicted caregiver satisfaction, and that less autonomous patients require more help, satisfaction with the heath care management of ASD at all health care settings might be increased if caregivers are provided with a better diagnostic process and are given better access to rehabilitation and post-diagnosis support services.

Further research that includes a nationally representative sample is needed to confirm these findings. A qualitative study should be conducted as an extension of this study in order to obtain an overall picture and clarify how the services provided meet caregivers’ needs and affect their satisfaction, thereby generating feedback about suggested improvements. 

## 5. Study Limitations

The findings in this study were based on caregivers’ reports. As such, inaccurate reporting might have biased our findings. The majority of the sample was derived from attendants of an intervention at government health care facilities. We thus did not include caregivers from private centers or those who left the intervention program, as doing so would have been too difficult. 

The current study was conducted in Kelantan, 96.0% of which are Malay residents [43]. Thus, we could not generalize the current findings to the entire population of Malaysia, but it can explain the needs of the Malay population in Kelantan and other states that shared the same culture and ethnicity. In this study, we intended to generalize the finding to those caregivers who attended and experience the ASD management at public health care. 

The sample sizes for primary and secondary care did not meet our calculated minimum sample size (n = 128). However, this did not substantially affect the validity of the analysis. Based on the rule of thumb for linear regression analysis, 10 subjects per variables (independent and dependent variables) are sufficient [44]. Based on this rule of thumb, the minimum sample sizes for each health care setting were n = 110 at primary care (10 independent variables with dummy variables and one dependent variable), n = 40 at secondary care (three independent variables and one dependent variable). These values exceeded the sample sizes for each care centers, which are n = 126 and n = 46 for primary and secondary care centers respectively. In addition, the confidence intervals for the estimated coefficients were reasonably narrow, which further justify this point.

In addition to that, the sampling method also introduces bias as those who actually visited different health care providers might give a different view on satisfaction level toward ASD management as compared to those only attended tertiary care.

Despite these limitations, this study provided a valuable picture of vulnerable families in need of proper care for their ASD children in Kelantan.

## 6. Conclusions

The current study described caregivers’ experiences, perceptions, and satisfaction throughout the process of attaining care for their children at different types of health care facilities in Kelantan. The study findings provide a valuable outline of families affected by ASD and current services for ASD in Kelantan. Taking into account the predictors of caregiving satisfaction found in the current study, we should primarily aim to increase ASD awareness among caregivers and health care professionals. In order to improve current services to suit caregivers’ needs, specific predictors should be considered by policymakers and health care professionals who are directly or indirectly involved in providing care for children with ASD and their families.

## Figures and Tables

**Table 1:** Socio-demographic characteristics of the caregivers (n = 227).

Variables	Frequency (%)	Mean (SD)
Age		38.91 (8.26)
Occupation		
Professional	89(39.2)	
Non-Professional	59(26.0)	
Housewife/Unemployed	79(34.8)	
Main Caregiver		
Mother	127(55.9)	
Father	19(8.4)	
Both	73(32.2)	
Others	8(3.5)	
Ethnicity		
Malay	210 (92.5)	
Non-Malay (Chinese, Siamese)	17 (7.5)	
Caregiver Education		
Secondary school and below	82(36.1)	
Diploma above	145(63.9)	
Household income (Ringgit Malaysia (RM))		
≤RM 2000	63(27.8)	
RM 2000 till less than RM 5000	88(38.8)	
RM 5000 till less than RM 8000	41(18.1)	
≥RM 8000	35(15.4)	
Problem with transportation		
Yes	26(11.5)	
No	201(88.5)	
Has medical problem		
Yes	11(4.8)	
No	216(95.2)	

**Table 2:** Socio-demographic characteristics of the ASD children (n = 227).

Variables	Frequency (%)	Mean (SD)
Age		7.45(3.54)
Gender		
Boy	188(82.8)	
Girl	39(17.20)	
ASD with comorbidity		
Yes	114(50.2)	
No	113(49.8)	
Age of child when caregiver start to show concern (year)		2.66(1.66)
Caregiver concern and worries when the child has:		
Speech delay		
Yes	212(93.4)	
No	15(6.6)	
Delay walking		
Yes	46(20.3)	
No	181(79.7)	
Social problem		
Yes	137 (60.4)	
No	90(39.6)	
Dislike changes		
Yes	84(37.0)	
No	143(63.0)	
Hyperactive child		
Yes	112(49.3)	
No	115(56.8)	
Learning disability		
Yes	98(43.2)	
No	129(56.8)	
Medical problem		
Yes	19(8.4)	
No	208(91.6)	
Hearing Problem		
Yes	34(15.0)	
No	195(85.0)	
Sensory Hypersensitivity		
Yes	84(37.0)	
No	143(63.0)	
Sleeping problem		
Yes	77(33.9)	
No	150(66.1)	
Does the child attend school?		
Not schooling	43 (18.9)	
Government school	67(29.5)	
Private school	117(51.6)	
Age of diagnosis with ASD (year)		4.98(1.85)
Age of child when caregiver seek help for first time (year)		3.72(2.08)

**Table 3:** Summary of the mean scores of satisfaction among caregivers of children with ASD at different health care facilities in Kelantan.

Item Describing Interpersonal Relationship with Professionals (Availability, Attitudes, Helpfulness, Supportiveness, Inclusion, and Decision Making)				Mean (SD)					
	Primary Care (*n* = 146)			Secondary Care (*n* = 46)			Tertiary Care (*n* = 227)		
	Each Item	Min, Max	Total Score	Each Item	Min, Max	Total Score	Each Item	Min, Max	Total Score
1. Overall, I was satisfied with the staff	2.46 (0.96)	1, 4	26.55 (7.16)	2.87 (0.65)	1, 4	30.06 (6.47)	3.04 (0.65)	0, 4	31.79 (6.25)
2. I was satisfied with the availability of the staff	2.35 (1.04)	0, 4		2.80 (0.86)	1, 4		3.04 (0.65)	0, 4	
3. I was satisfied with the way the staff helped me understand my child’s problems	2.13 (1.11)	0, 4		2.65 (0.90)	1, 4		2.89 (0.86)	0, 4	
4. I was satisfied with the convenience of appointments with the staff	2.60 (0.96)	0, 4		2.76 (0.97)	1, 4		2.56 (1.12)	0, 4	
5. I was satisfied with the caring and concern the staff showed for my child	2.40 (1.07)	1, 4		2.65 (0.88)	1, 4		2.94 (0.83)	0, 4	
6. I was satisfied with how the staff treated me with respect	2.71 (0.88)	1, 4		2.76 (0.82)	1, 4		3.00 (0.73)	0, 4	
7. I was satisfied with how the staff listened to what I had to say	2.10 (1.11)	1, 4		2.61 (0.98)	1, 4		2.88 (0.87)	0, 4	
8. I was satisfied with how the staff kept me informed about changes in the care of my child	1.96 (1.06)	0, 4		2.57 (0.98)	1, 4		2.81 (0.89)	0, 4	
9. I was satisfied with how the staff helped me find the services my child needed	2.66 (0.82)	1, 4		2.89 (0.64)	1, 4		2.96 (0.76)	0, 4	
10. I was satisfied with how the staff included me in decision making about my child’s treatment	2.67 (0.83)	1, 4		2.80 (0.75)	1, 4		2.85 (0.82)	0, 4	
11. I was satisfied with the support I received from the staff	2.51 (0.98)	1, 4		2.70 (0.84)	1, 4		2.07 (1.14)	0, 4	

**Table 4:** Predictors of caregivers’ satisfaction with the health care management of children with ASD at primary care in Kelantan (*n* = 126).

Variables	Mean Satisfaction Score (SD)	Simple Linear Regression		Multiple Linear Regression	
		*b*^a^ (95% CI)	*p*-value	*b*^a^ (95% CI)	*p*-value
Household income (RM)					
<2000	26.58 (1.20)	0		0	
2000–5000	26.49 (0.94)	−0.12 (−2.98, 2.74)	0.942	1.64 (−0.71,3.98)	0.174
5001–8000	28.47 (1.42)	1.89 (1.78, 5.56)	0.321	3.34 (0.32, 6.37)	0.028
>8000	22.70 (1.69)	−3.89 (−8.75, 0.98)	0.123	0.32 (−3.86,4.49)	0.882
Presence of sensory hypersensitivity					
No	27.76 (0.69)	0			
Yes	24.63 (1.18)	−3.13 (−5.64, −0.62)	0.016	−2.33 (−4.47, −0.19)	0.035
Consulted doctors (for diagnosis confirmation)					
3 times or less	27.90 (0.84)	0			
More than 3 times	24.74 (0.94)	−2.56 (−5.05, −0.08)	0.046	−2.25 (−4.39, −0.12)	0.041
Waiting time					
Less than 30 min	31.82 (1.25)	0		0	
30 min to 1 h	26.62 (0.81)	−5.19 (−8.32, −2.08)	0.001	−2.51 (−5.42,0.39)	0.091
1 h to 2 h	26.26 (1.23)	−5.56 (−9.16, −1.96)	0.003	−2.11 (−5.55,1.33)	0.232
More than 2 h	19.50 (1.82)	−12.318 (−16.44, −8.19)	<0.001	−6.78 (−10.88, 2.40)	0.001
Satisfied with waiting time					
No	23.52 (0.79)	0			
Yes	30.21 (0.81)	6.69 (4.46, 8.92)	<0.001	3.88 (1.60, 6.22)	<0.001
Satisfied with consultation time with doctors					
No	23.83 (0.78)	0			
Yes	29.53 (0.88)	5.70 (3.39, 8.00)	<0.001	2.71 (0.36, 5.06)	0.025

^a^ Crude regression coefficient; ^b^ Adjusted regression coefficient. Forward multiple linear regression method applied. Model assumptions were fulfilled. There were 2 interactions among the independent variables. No multicollinearity was detected. Coefficient of determinants, R^2^ (adjusted) = 41.56%.

**Table 5:** Predictors of caregivers’ satisfaction with the health care management of children with ASD at secondary care in Kelantan (*n* = 46).

Variables	Mean Satisfaction Score (SD)	Simple Linear Regression		Multiple Linear Regression	
		*b*^a^ (95% CI)	*p*-value	*b*^a^ (95% CI)	*p*-value
Caregiver education					
Secondary school or below	32.72 (1.19)	0			
Diploma or above	26.80 (1.26)	−5.93(-9.35, −2.51)	<0.001	−5.99(−8.76, −3.22)	<0.001
Satisfied with occupational therapy appointment					
No	24.87 (1.70)	0			
Yes	31.78 (0.96)	6.92 (3.34, 10.49)	<0.001	4.05 (0.99, 7.12)	0.012
Satisfied with consultation time with doctors					
No	25.50 1.68)	0			
Yes	31.68 (1.00)	6.18 (2.56, 9.79)	<0.001	5.93 (2.94, 8.91)	<0.001

^a^ Crude regression coefficient; ^b^ Adjusted regression coefficient. Forward multiple linear regression method applied. Model assumptions were fulfilled. There was no interaction among the independent variables. No multicollinearity was detected. Coefficient of determinants, R^2^ (adjusted) = 50.65%.

**Table 6:** Predictors of caregivers’ satisfaction with the health care management of children with ASD at tertiary care (*n* = 227).

Variables	Mean Satisfaction Score (SD)	Simple Linear Regression		Multiple Linear Regression	
		*b*^a^ (95% CI)	*p*-value	*b*^a^ (95% CI)	*p*-value
Caregiver with medical problems					
No	31.25 (0.34)	0	0.013		
Yes	26.45 (3.37)	−4.80 (−8.55, −1.05)		−6.09 (−9.32, −2.85)	<0.001
Presence of sleeping problems					
No	30.71 (0.54)	0			
Yes	31.64 (0.64)	0.92 (−0.79, 2.65)	0.291	1.65 (0.09, 3.11)	0.035
Offered support group post-diagnosis					
No	30.79 (0.40)	0			
Yes	33.94 (2.47)	3.15 (0.08, 6.23)	0.046	3.11 (0.48, 5.73)	0.021
Frequency of occupational therapy					
Once monthly or less	30.87 (0.41)	0			
Twice monthly or more	36.67 (4.04)	5.79 (0.76, 10.83)	0.025	−5.23 (1.00, 9.46)	0.016
Satisfied with frequency of appointments with speech therapist					
No	29.70 (0.61)	0			
Yes	32.62 (0.53)	2.92 (−9.25, 7.84)	<0.001	1.66 (0.20, 3.11)	0.026
Satisfied with frequency of occupational therapy appointments					
No	26.14 (1.89)	0			
Yes	31.52 (0.40)	5.38 (2.65, 8.11)	<0.001	3.82 (1.35, 6.31)	0.003
Satisfied with waiting time					
No	29.14 (0.54)	0			
Yes	32.42 (0.57)	3.28 (1.68, 4.87)	<0.001	2.55 (1.12, 3.98)	<0.001
Satisfied with doctor’s knowledge and experience					
No	26.53 (0.80)	0			
Yes	31.98 (0.45)	5.46 (3.43, 7.47)	<0.001	4.39 (2.51, 6.29)	<0.001

^a^ Crude regression coefficient; ^b^ Adjusted regression coefficient. Forward multiple linear regression method applied. Model assumptions were fulfilled. There were 2 interactions among the independent variables. No multicollinearity was detected. Coefficient of determinants, R^2^ (adjusted) = 32.92%.
